# Supplementary material for: Integrated analysis of the aqueous humor microbiome and lens capsule transcriptome in high myopia cataract: a pilot study
Source: Front Med (Lausanne). 2026 Jun 16;13:1845205. doi: 10.3389/fmed.2026.1845205 (PMC13314463; doi:10.3389/fmed.2026.1845205)
Supplement: Supplementary file 3 [file Data_Sheet_1.zip › 3.Beta_Diversity/PCA/C372089/PCA_2D.pdf]

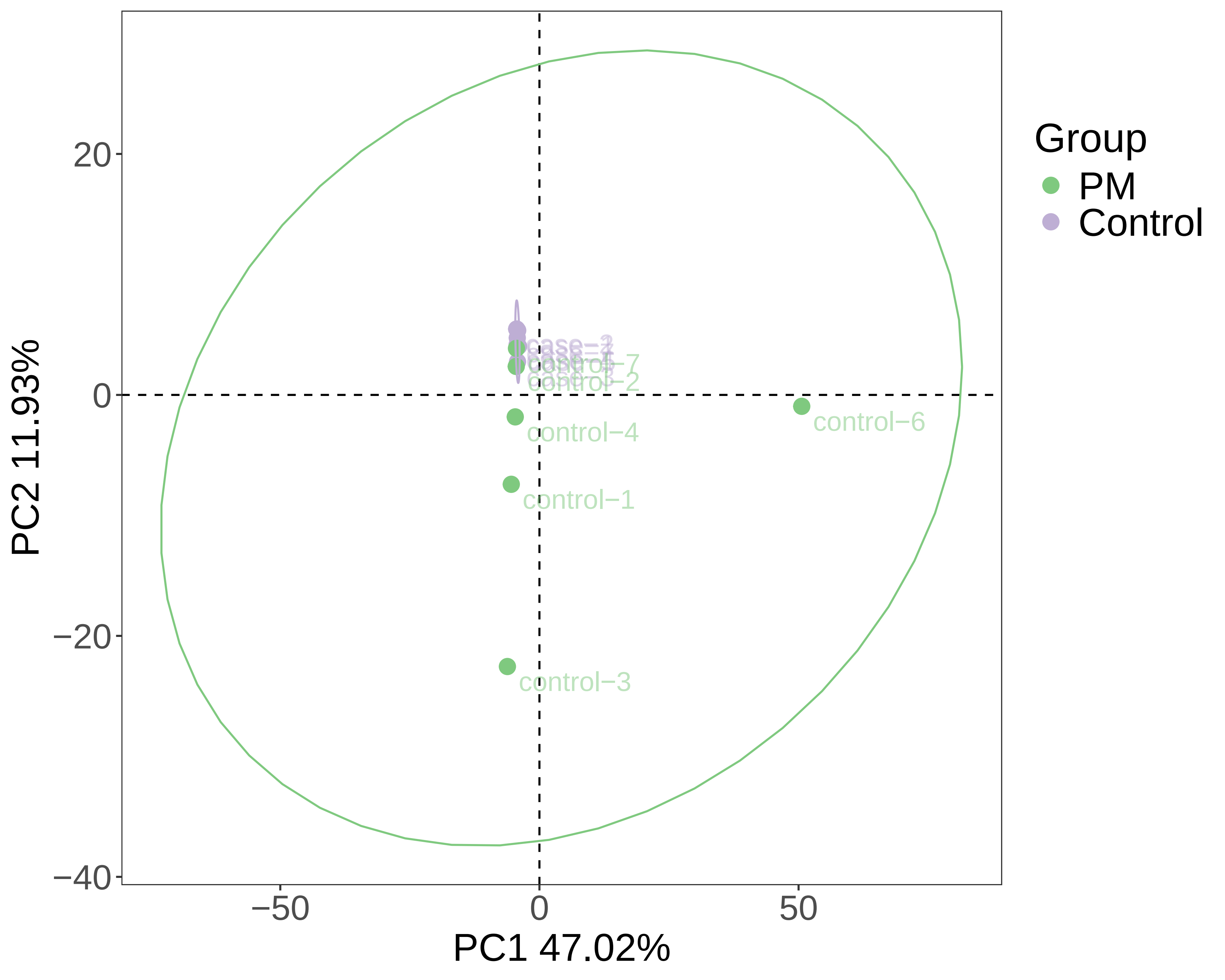

Group
PM
Control
PC1 47.02%
PC2 11.93%
20
0
−20
−40
−50
0
50
control−7
control−2
control−4
control−6
control−1
control−3
